# 固相支撑液液萃取-超高效液相色谱-串联质谱法测定人体尿液中17种双酚类化合物

**DOI:** 10.3724/SP.J.1123.2024.11032

**Published:** 2025-09-08

**Authors:** Yu’e JIN, Lange ZHANG, Jingxian ZHOU, Jinjing MA, Lili YUAN, Ping XIAO, Guoquan WANG

**Affiliations:** 1.上海市疾病预防控制中心，化学品毒性检定所，国家环境保护新型污染物环境健康影响评价重点实验室，上海 200336; 1. Division of Chemical Toxicity and Safety Assessment，Shanghai Municipal Center for Disease Control and Prevention，Key Laboratory of Environmental Health Impact Assessment of New Pollutants in National Environmental Protection，Shanghai 200336，China; 2.上海交通大学医学院附属新华医院环境与儿童健康教育部与上海市重点实验室，上海 200092; 2. Ministry of Education-Shanghai Key Laboratory of Children's Environmental Health，Xinhua Hospital，Shanghai Jiao Tong University School of Medicine，Shanghai 200092，China

**Keywords:** 固相支撑液液萃取, 超高效液相色谱-串联质谱, 双酚类化合物, 人体尿液, solid supported liquid-liquid extraction （SLE）, ultra-high performance liquid chromatography-tandem mass spectrometry （UHPLC-MS/MS）, bisphenol compounds （BPs）, human urine

## Abstract

双酚A（BPA）及其类似物统称为双酚类化合物（BPs），这类物质主要用于生产聚碳酸酯塑料和环氧树脂，因此广泛分布于各类环境介质、人体组织及代谢产物中。现有研究表明，BPs会对神经系统、生殖系统、免疫系统和代谢系统产生不良影响。本文将固相支撑液液萃取（SLE）与超高效液相色谱-串联质谱（UHPLC-MS/MS）技术结合，建立了一种人体尿液中17种BPs的高通量测定方法。样品经酶解处理后，依次进行全自动SLE净化、氮吹浓缩和复溶等步骤。使用CAPCELL PAK ADME色谱柱（100 mm×2.1 mm，2 μm）进行分离，以0.05 mmol/L氟化铵水溶液和0.05 mmol/L氟化铵甲醇溶液作为流动相进行梯度洗脱。质谱检测采用电喷雾电离（ESI）负离子扫描模式，在多反应监测（MRM）模式下进行，通过保留时间及离子丰度比进行定性，内标法进行定量。在优化的条件下，17种BPs可实现有效分离。17种BPs在对应的质量浓度范围内线性关系良好，相关系数（*r*）≥0.998 6，检出限（LOD）为0.002~0.489 μg/L，定量限（LOQ）为0.005~0.986 μg/L。选取低本底含量的儿童混合尿样为基质，在低、中、高3个加标水平下进行加标回收试验，结果表明，17种BPs的回收率为61.1%~121.7%，日内精密度为1.3%~11.2%，日间精密度为3.7%~19.0%。采用本方法对50份随机尿样进行测定，结果表明，共有11种BPs被检出，其中双酚S（BPS）和BPA的检出率最高，分别为98.0%和86.0%，检出水平中位值分别为0.075 μg/L和0.829 μg/L。本文所建方法操作简便、灵敏可靠，适用于人体尿液中17种BPs的快速定量分析，可为人群BPs暴露风险评估提供有效的技术支撑。

## Abstract (en)

Bisphenol A （BPA） and its analogs are collectively termed bisphenol compounds （BPs）， which are predominantly utilized in the manufacturing of polycarbonate plastics and epoxy resins. BPs are ubiquitous in diverse environmental matrices， human tissues， and metabolic products. Extensive research has demonstrated that BPs exert adverse effects on the nervous， reproductive， immune， and metabolic systems. After exposure in humans， BPs are primarily excreted in urine. Consequently， the development of efficient and robust analytical methods for BPs quantification in urine is essential for assessing population exposure levels. In this study， solid supported liquid-liquid extraction （SLE） was combined with ultra-high performance liquid chromatography-tandem mass spectrometry （UHPLC-MS/MS） technology to establish a high-throughput determination method for 17 BPs in human urine. After enzymatic hydrolysis treatment of the samples， the steps of fully automatic SLE purification， nitrogen blowing concentration and redissolution were carried out successively. Separation was performed using a CAPCELL PAK ADME chromatography column （100 mm×2.1 mm， 2 μm）， and gradient elution was carried out with 0.05 mmol/L ammonium fluoride aqueous solution and 0.05 mmol/L ammonium fluoride methanol solution as the mobile phases. MS detection was carried out using the electrospray ionization （ESI） negative ion scanning mode under the multi-reaction monitoring （MRM） mode. Qualitative analysis was conducted based on retention time and ion abundance ratio， and quantitative analysis was performed using the internal standard method. Under the optimized conditions， 17 BPs can be effectively separated. The linear relationships of the 17 BPs within the corresponding mass concentration ranges were good， and the correlation coefficients （*r*） were ≥0.998 6， the limits of detection （LODs） and quantification （LOQ） were 0.002-0.489 μg/L and 0.005-0.986 μg/L， respectively. Children’s mixed urine samples with low background content were selected as the matrix， and then spiked recovery tests were conducted at three spiked levels （low， medium and high）. The results showed that the recoveries of 17 BPs were 61.1%-121.7%， the intra-day RSDs were 1.3%-11.2%， and the inter-day RSDs were 3.7%-19.0%. This method was used to determine 50 random urine samples， and the results showed that a total of 11 BPs were detected. Among them， bisphenol S （BPS） and BPA had the highest detection rates， which were 98.0% and 86.0% respectively， and the median detection levels were 0.075 μg/L and 0.829 μg/L respectively. This method is simple to operate， sensitive and reliable. It is suitable for the rapid quantitative analysis of 17 BPs in human urine and can provide effective technical support for the risk assessment of BPs exposure in the population.

双酚类化合物（bisphenol compounds，BPs）是一类由两个酚环通过碳桥或其他化学结构连接而成的有机化合物^［1］^。由BPs制成的合成聚合物具有优异的电绝缘性、机械性能及热稳定性，因此被广泛应用于食品和饮料包装容器、婴儿奶瓶、医疗设备及电子设备等消费品中^［2-4］^。用于收据和标签的热敏纸是继塑料制品之后人类暴露于双酚A（bisphenol A，BPA）的第二大来源^［5］^。据估算，工业领域应用最广泛的BPA全球年产量约为60亿磅，其中有65%和30%分别用于聚碳酸酯塑料和环氧树脂的生产^［6，7］^。BPA作为一种内分泌干扰化学物质，具有类雌激素和抗雄激素活性，可对神经系统、生殖系统、免疫系统及代谢系统产生不良影响^［8］^。自2015年起，欧盟已全面禁止在食品接触材料中使用BPA^［7］^。我国亦规定食品或食品模拟物中BPA的迁移量不得超过0.6 mg/kg^［9］^。然而，对BPA的使用限制推动了其类似物的研发与应用，工业生产中常见的类似物包括双酚S（bisphenol S， BPS）、双酚F（bisphenol F， BPF）、双酚AF（bisphenol AF， BPAF）、四溴双酚A（tetrabromobisphenol A， TBBPA）及双酚Z（bisphenol Z， BPZ）等，这些物质广泛应用于塑料、光纤及阻燃剂等领域。进一步研究表明，由于结构相似性，这些BPA类似物同样具有与BPA相当的内分泌干扰效应^［10］^。

大规模的生产与使用增加了BPs的环境污染途径。有研究表明，BPs已广泛分布于水体、沉积物、土壤及大气等生态介质中^［11］^。饮食摄入是人类接触BPs的主要途径，而通过个人护理品、热敏纸及钞票等生活用品的皮肤接触暴露亦不可忽视^［12］^。BPs进入人体后会迅速被肠胃吸收，其中大部分在肝脏和肠道代谢为葡萄糖醛酸结合物，并通过尿液排泄，其生物半衰期约为6 h^［10，13］^。在中国成年人群中，86%的血浆样本可检出BPs，总含量范围为未检出（ND）~5.6 μg/L^［14］^。此外，BPs亦在母乳^［15］^、汗液^［16］^、羊水^［17］^及胎盘^［18］^等生物基质中被检出。鉴于尿液样本具有采样便捷、无创及易于操作等优势，常通过测定尿液中总的BPs（游离态与结合态）含量来综合评估人体对BPs的多途径暴露水平。

BPs在生物样本中通常以痕量水平存在。目前常用的前处理技术包括固相萃取（solid-phase extraction， SPE）^［19］^、液液萃取（liquid-liquid extraction， LLE）^［20］^及QuEChERS^［21］^等。其中，SPE操作流程复杂且耗时较长；LLE虽操作简便，但需消耗大量有机溶剂，且在提取过程中易发生乳化现象；QuEChERS方法具有快速、简便及成本较低的优势，但对复杂生物基质的净化能力有限，存在提取效率低、目标物损失大等局限性。近年来，固相支撑液液萃取（SLE）作为一种新兴的高效前处理方法受到关注。该技术以高比表面积、化学惰性的多孔硅藻土为液液分配载体，通过与水相样本的相互作用使目标分析物吸附于载体表面，随后利用不相溶的有机溶剂洗脱目标分析物，而盐类及极性干扰物等基质成分则被保留在载体中。SLE技术兼具快速、简便的特点，有效克服了传统方法易乳化、耗时长及溶剂用量大等缺陷。针对内暴露分析需求，SLE通过物理吸附分离机制可最大限度地保留痕量分析物，提升回收率，同时避免QuEChERS方法中分散固相吸附剂对低水平目标物的不可逆吸附。此外，SLE与自动化工作站兼容性良好，可实现高通量样本处理，更契合生物监测中大样本量检测的实际需求。目前，该技术已逐步应用于人体样本中BPs、对羟基苯甲酸酯及多环芳烃代谢物等有机污染物的提取与净化^［22，23］^。

本研究以17种常见的BPs为研究对象，通过优化SLE条件，对尿液进行萃取和净化处理，并选取合适的色谱柱、洗脱梯度以及质谱参数，建立了一种可同时测定人体尿液中17种BPs的定量分析方法。该方法有助于进一步推动该类化合物生物监测工作的开展，为评估其暴露水平和健康风险奠定基础。

## 1 实验部分

### 1.1 仪器、试剂与材料

Agilent 1290 Infinity II超高效液相色谱仪（美国Agilent公司）；Qtrap 6500+三重四极杆线性离子阱复合型质谱仪（美国AB Sciex公司）；Biotage^®^ Extrahera全自动SPE净化工作站、TurboVap多功能全自动样品浓缩仪、1 mL ISOLUTE^®^ SLE+固相支撑液液萃取小柱（瑞典Biotage公司）；16 mm×75 mm样品管、样品收集管（美国DWK LIFE SCIENCES公司）VORTEX GENIUS 3旋涡混匀器（德国IKA公司）；SHA-C恒温振荡器（国华（常州）仪器制造有限公司）；Supelco^®^ Visiprep DL固相提取系统（美国Supelco公司）；Milli-Q超纯水机（美国Millipore公司）。

甲醇（HPLC级，北京迪马科技有限公司）；乙酸乙酯（EA，HPLC级，美国Fisher公司）；甲基叔丁基醚（MTBE，HPLC级）、*β*-葡萄糖苷酸酶/芳基硫酸酯酶（30 U/60 U，提取自蜗牛体内，德国Merck公司）；异丙醇（IPA，HPLC级，美国Tedia公司）；乙酸铵（98%，上海凌峰化学试剂有限公司）；乙酸（质谱级）、氨水（25%~28%，上海安谱实验科技股份有限公司）；氟化铵（≥98%，美国Fluka公司）；质谱水（LC-MS级，上海安谱实验科技股份有限公司）。

15种BPs的混合标准溶液（质量浓度为100 μg/mL，溶剂为甲醇）购自天津阿尔塔科技有限公司，具体包括BPA、BPAF、双酚AP（BPAP）、双酚B（BPB）、双酚C（BPC）、BPF、双酚P（BPP）、BPS、BPZ、双酚BP（BPBP）、双酚C2（BPC2）、双酚E（BPE）、双酚G（BPG）、双酚M（BPM）和双酚pH（BPPH）；四溴双酚A（TBBPA）（纯度>98.3%，北京曼哈格生物科技有限公司），四氯双酚A（TCBPA）（纯度>99.6%，上海安谱实验科技股份有限公司）。10种同位素内标：BPA-D_4_和BPS-D_8_（纯度98%）购自加拿大CDN ISOTopes公司，BPAF-^13^C_12_（纯度97%）购自加拿大TRC公司，BPF-^13^C_6_（纯度93%）购自德国Dr. Ehrenstorfer公司，TBBPA-D_4_（纯度>98%）购自北京曼哈格生物科技有限公司，BPB-D_8_、BPE-^13^C_6_、BPP-^13^C_4_、BPZ-^13^C_12_和BPAP-D_5_（纯度>98%）购自天津阿尔塔科技有限公司。标准参考物质^®^（SRM）3672和3673购自美国国家标准与技术研究院（NIST）。

### 1.2 溶液的配制

2种BPs（TBBPA和TCBPA）固体标准品和10种同位素内标分别使用甲醇溶解，配制成质量浓度为1 g/L的单标储备液和内标储备液；分别量取适量的2种BPs单标储备液和15种BPs混合标准溶液（100 μg/mL），用50%甲醇水溶液混合稀释，配制成质量浓度为250 μg/L的17种BPs混合标准溶液；用50%甲醇水溶液混合稀释10种内标储备液，配制成质量浓度均为1 mg/L的内标混合溶液。临用前用50%甲醇水溶液逐级稀释混合标准溶液，并加入适量的内标混合溶液，配制成质量浓度分别为0.05、0.1、0.2、0.4、1、2、4、10、20和40 μg/L的混合标准工作溶液，其中内标的质量浓度均为10 μg/L。

### 1.3 样品前处理

尿液样品取自在上海市人类精子库中捐献精子的志愿者。本研究已获得上海交通大学医学院附属新华医院医学伦理委员会批准，项目伦理批件号为XHEC-C-2020-114-1。

尿液样品采集完成后，于-80 ℃条件下储存。在测定前，将尿液样品置于4 ℃冰箱中充分解冻，之后取出并使其恢复至室温。随后，将尿液样品涡旋混匀，在4 000 r/min下离心5 min，准确吸取750 μL上清液加入到已预先加入30 μL内标混合溶液（50 μg/L）的玻璃试管中，再向试管中加入100 μL乙酸-乙酸铵缓冲溶液（pH 5.2）和20 μL *β*-葡萄糖苷酸酶/芳基硫酸酯酶，充分混匀后置于37 ℃水浴恒温振荡器中，避光酶解16 h以上。16 h后取出样品并冷却至室温，加入110 μL氨水待用。

### 1.4 SLE净化

将样品管、SLE小柱和样品收集管分别安装到全自动SPE净化工作站的样品盘、SPE支架和收集管支架上，然后自动运行提前设置好的SLE过柱程序。过柱步骤如下：用移液枪快速吸放样品3次，接着从样品管中吸取1 mL样品转移至SLE小柱上，用20 kPa的氮气加压10 s，待样品全部被SLE小柱的填料吸附后，静置5 min；随后加入2 mL乙酸乙酯，并分别用20、30和100 kPa的氮气依次加压2、1和1 min进行洗脱；收集全部洗脱液，置于氮吹仪中，在40 ℃下用氮气吹干；最后用150 μL 50%甲醇水溶液复溶，超声处理后涡旋混匀，将样品转移至带衬管的玻璃进样小瓶中，进样分析。

### 1.5 仪器分析条件

#### 1.5.1 色谱条件

色谱柱：CAPCELL PAK ADME色谱柱（100 mm×2.1 mm，2 μm）；柱温：40 ℃；样品室温度：10 ℃；运行时间：13 min。流动相A为0.05 mmol/L氟化铵水溶液，流动相B为0.05 mmol/L氟化铵甲醇溶液，流速0.4 mL/min，进样量10 μL。梯度洗脱程序：0 min，40%B；0~5.0 min，线性增加至70%B；5.0~8.5 min，线性增加至80%B；8.5~8.7 min，线性增加至90%B；8.7~10.0 min，保持90%B；10.0~10.2 min，线性降低至40%B；10.2~13.0 min，保持40%B。

#### 1.5.2 质谱条件

离子源：ESI源，负离子模式；气帘气压力：2.76×10^5^ Pa；碰撞气压力：Medium；喷雾电压：-4 500 V；雾化温度：500 ℃；喷雾气压力：3.45×10^5^ Pa；辅助加热气压力：3.45×10^5^ Pa。采用多反应监测（MRM）模式进行扫描，17种BPs及10种同位素内标的保留时间和监测离子对等质谱参数见表1。

**表1:** 17种BPs及10种同位素内标的保留时间和质谱参数

Analyte	Retention time/min	Precursor ion （*m/z*）	Product ions （*m/z*）	CEs/eV	DPs/V
Bisphenol S （BPS）	3.45	249	108^*^， 92	-35， -38	-92
Bisphenol F （BPF）	4.53	199	93^*^， 105	-26， -27	-86
Bisphenol E （BPE）	5.24	213	198^*^， 197	-23， -34	-56
Bisphenol A （BPA）	5.84	227	212^*^， 133	-24， -33	-68
Bisphenol B （BPB）	6.51	241	212^*^， 211	-24， -32	-90
Bisphenol AF （BPAF）	6.81	335	265^*^， 197	-30， -51	-70
Bisphenol C2 （BPC2）	7.00	279， 281	35^*^， 37	-55， -55	-96
Bisphenol C （BPC）	7.25	255	147^*^， 239	-34， -33	-90
Bisphenol Z （BPZ）	7.50	267	173^*^， 223	-35， -42	-132
Bisphenol AP （BPAP）	7.61	289	274^*^， 211	-27，-34	-86
Bisphenol M （BPM）	9.37	345	133^*^， 329	-41， -45	-137
Bisphenol G （BPG）	9.38	311	295^*^， 175	-40， -43	-130
Bisphenol BP （BPBP）	9.48	351	273^*^， 274	-34， -31	-92
Bisphenol P （BPP）	9.61	345	330^*^， 133	-37， -43	-123
Tetrachlorobisphenol A （TCBPA）	9.65	365	314^*^， 286	-34， -36	-164
Tetrabromobisphenol A （TBBPA）	10.09	543	446^*^， 291	-44， -50	-112
Bisphenol PH （BPPH）	10.41	379	209^*^， 363	-47， -50	-148
BPA-D_4_	3.42	231	216^*^， 135	-24， -33	-74
BPS-D_8_	3.45	257	112^*^， 96	-35， -42	-90
BPF-^13^C_6_	4.53	205	99^*^， 93	-29， -29	-80
BPE-^13^C_6_	5.24	219	204^*^， 203	-23， -32	-54
BPB-D_8_	6.51	249	219^*^， 220	-34， -24	-96
BPAF-^13^C_12_	6.81	347	277^*^， 208	-29， -51	-60
BPZ-^13^C_12_	7.50	279	179^*^， 235	-38， -41	-118
BPAP-D_5_	7.61	294	279^*^， 211	-29， -38	-40
BPP-^13^C_4_	9.61	349	333^*^， 135	-35， -45	-85
TBBPA-D_4_	10.09	547	424^*^， 452	-55， -45	-132

* Quantitative ion； CEs： collision energies； DPs： declustering potentials.

### 1.6 质量控制

为降低试剂和材料中的本底值，在实验过程中采取了以下措施：使用高纯度有机溶剂和超纯水；试管、进样瓶等材料均选用玻璃制品；试剂均在具塞玻璃容器中配制，且玻璃容器需事先用超纯水和高纯度甲醇多次冲洗。在前处理阶段，以等体积的质谱水作为基质，与尿液样本同步处理，制备全流程空白样品，以此监测实验室环境或其他来源可能引入的污染。为评估仪器背景情况以及检测是否存在仪器残留，设定每进样10个样品后插入1个空白甲醇溶液。实验中采用10种BPs同位素内标，用于评估尿液样本中目标化合物的提取效果。鉴于复溶液经实验室常用的3种材质（醋酸纤维素、聚四氟乙烯和聚偏二氟乙烯）的0.22 μm微孔滤膜过滤后，均检测出一定水平的BPA，且SLE小柱净化过程本身已具备一定的过滤作用，因此本方法最终确定样品复溶液直接上机测定。

## 2 结果与讨论

### 2.1 仪器条件优化

#### 2.1.1 质谱条件的优化

将17种BPs的混合标准溶液（50 μg/L）注入离子源，根据其化学电离特性，采用ESI源负离子模式进行一级质谱分析，以确定目标化合物准分子离子的质量数；随后，通过二级质谱分析获取准分子离子的碎片离子信息，选取二级质谱模式下响应强度较高的特征子离子碎片为定量离子和定性离子，其中响应强度最高的离子作为定量离子。在MRM模式下对上述离子对的去簇电压（DP）和碰撞能量（CE）进行优化，同时也对10种同位素内标的质谱条件展开优化。优化后的17种BPs及10种同位素内标的质谱参数如表1所示。

#### 2.1.2 色谱条件的优化

17种BPs的正辛醇/水分配系数（log *P*）均大于0，且彼此间数值接近，它们属于弱极性到非极性且性质较为相近的一组化合物^［24］^。鉴于此，本研究选取了3种常规的UHPLC柱（ACQUITY UHPLC Premier BEH C18柱（100 mm×2.1 mm，1.7 μm）、ACQUITY UHPLC HSS T3柱（100 mm×2.1 mm，1.8 μm）和CAPCELL PAK ADME柱（100 mm×2.1 mm，2 μm））进行分离优化实验。其中，Premier BEH C18色谱柱在传统C18色谱柱的基础上，采用了MaxPeak高性能表面硬件技术，该技术可减少分析物与色谱柱表面的相互作用，进而改善峰形，增强信号强度，适用于大多数化合物的分离。HSS T3色谱柱则是在C18键合相的基础上增加了极性基团修饰，使其能够耐受更高比例的水相，因此更适合分离极性较强的化合物。ADME色谱柱键合了金刚烷胺基团，其表面特性和分离能力与直链碳链色谱柱不同，对极性化合物和疏水性化合物均具有良好的保留和分离效果。实验发现，在相同的洗脱梯度条件下，极性最强的BPS在HSS T3色谱柱上几乎无法保留。在相对复杂的生物样品基质中，BPS必然会受到样品中杂质的干扰，进而影响对目标物的准确定性分析。为此，本研究对Premier BEH C18色谱柱和ADME色谱柱的梯度洗脱程序进行了优化。当将初始流动相中有机相的比例从65%调整为40%，并结合洗脱梯度的相应调整后，这两种色谱柱对BPS的保留效果均明显增强。具体而言，BPS在Premier BEH C18柱和ADME柱上的保留时间分别推迟至1.73 min和3.45 min。尽管大多数化合物在Premier BEH C18柱上的分离效果与ADME柱相近，但Premier BEH C18柱未能实现BPP和BPM这对同分异构体的基本分离，这可能会干扰二者在复杂基质中的准确定量。相比之下，ADME色谱柱能够在13 min内实现17种BPs的有效分离，且峰形良好。因此，本方法最终选择CAPCELL PAK ADME色谱柱（100 mm×2.1 mm，2 μm）。

在确定色谱柱后，实验对流动相的组成进行了优化，其中有机相包括甲醇、乙腈和甲醇-乙腈（1∶1，v/v），水相包括超纯水、0.1%氨水溶液和0.05 mmol/L氟化铵水溶液。实验结果表明，当水相采用超纯水且有机相选用甲醇时，17种BPs均能实现有效分离；而使用乙腈和甲醇-乙腈（1∶1，v/v）作为有机相时，17种BPs的分离效果不佳。因此，确定流动相中的有机相为甲醇。在确定有机相后对3种水相进行优化，结果发现，17种BPs在0.05 mmol/L氟化铵水溶液中的峰形及响应强度表现最佳。实验进一步对比了不同浓度（0.05、0.1和0.5 mmol/L）的氟化铵水溶液对目标物响应强度和分离效果的影响。结果表明，当采用0.05 mmol/L氟化铵水溶液作为水相时，17种BPs的响应值和分离效果达到最优。综合上述结果，为确保在梯度运行过程中，流动相比例的改变不会影响氟化铵的添加浓度，在有机相甲醇中同样添加相同浓度的氟化铵。最终，本方法采用0.05 mmol/L氟化铵水溶液和0.05 mmol/L氟化铵甲醇溶液作为流动相。在最佳仪器分析条件下，17种BPs的MRM色谱图见图1。

**图1:**
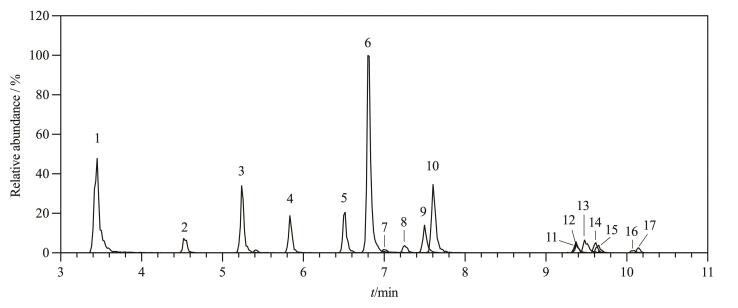
17种BPs的MRM色谱图 Peak identifications： 1. BPS； 2. BPF； 3. BPE； 4. BPA； 5. BPB； 6. BPAF； 7. BPC2； 8. BPC； 9. BPZ； 10. BPAP； 11. BPM； 12. BPG； 13. BPBP； 14. BPP； 15. TCBPA； 16. TBBPA； 17. BPPH.

### 2.2 SLE条件的优化

#### 2.2.1 上样溶液中氨水的体积

BPs是一类偏碱性化合物（酸度系数（p*K*_a_）为7.55~10.80），在过SLE柱前加入少量的碱性溶液（即提高上样溶液的pH值）可促进BPs在SLE填料中的分配及洗脱，从而获得更高的回收率。本研究使用氨水来调节上样溶液的pH值，以空白加标（50 μg/L）的混合尿样为基质，考察了不同体积（0、70、80、90、100、110和120 μL）的氨水对17种目标化合物绝对回收率（前处理后样品中目标化合物的峰面积/相同质量浓度标准品的峰面积）的影响。结果表明，与其他氨水添加量相比，当氨水添加量为110 μL时（上样溶液pH约为9.24），有9种BPs的绝对回收率从62.9%~90.2%提高至75.3%~97.0%，同时17种BPs的绝对回收率平均值也达到最高（80.8%），因此实验选择在前处理过程中添加110 μL氨水来调节上样溶液的pH。

#### 2.2.2 洗脱溶剂种类

BPs的结构中均含有两个或两个以上的苯环，其水溶性相对较低。本实验以绝对回收率为指标，对比了6种洗脱溶剂（EA、含5% IPA的EA、MTBE、含5% IPA的MTBE、EA-MTBE（1∶1，v/v）和含5% IPA的EA-MTBE（1∶1，v/v））对17种BPs的洗脱效果，其中洗脱溶剂的体积均为4 mL。结果如图2所示，用EA和含5% IPA的EA进行洗脱时，大部分目标化合物的绝对回收率相差不大，但对于BPS、BPC、BPZ、BPC2和BPAP这5种BPs，用EA洗脱所得到的绝对回收率比含5% IPA的EA提高了6.0%~21.5%。当采用另外4种洗脱溶剂（MTBE、含5% IPA的MTBE、EA-MTBE（1∶1，v/v）和含5% IPA的EA-MTBE（1∶1，v/v））进行洗脱时，除BPS外，其他16种目标化合物的绝对回收率均低于使用EA洗脱时所得到的绝对回收率。因此，本实验最终选择EA作为洗脱溶剂。

**图2:**
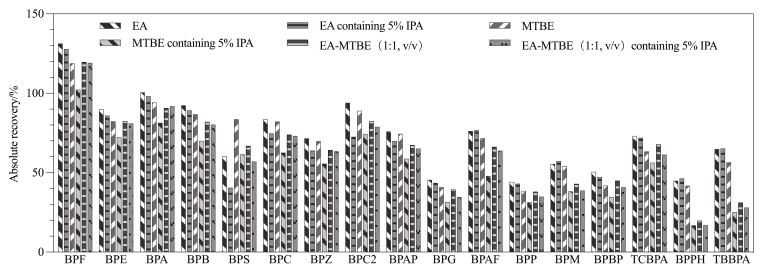
不同洗脱溶剂对17种BPs绝对回收率的影响

#### 2.2.3 洗脱溶剂体积

本方法以绝对回收率为指标，进一步考察了不同体积（1、2、3、4、5、6 mL）的EA对17种BPs洗脱效果的影响。结果表明，当洗脱溶剂体积为1 mL时，各目标化合物洗脱不完全，其中5种BPs的绝对回收率小于36.1%；当使用2~6 mL EA进行洗脱时，大部分目标化合物的绝对回收率均有了明显提升，且总体相差不大，但使用2 mL EA洗脱时，17种BPs绝对回收率的平均值最高（69.4%），其中BPAF、BPB和BPC的绝对回收率增加了19.1%、17.3%和16.5%。此外，采用2 mL EA洗脱不仅有机溶剂用量更少、更环保，还能在尽可能降低实验成本和空白本底值的同时，缩短氮吹浓缩所需的时间。因此，实验确定洗脱溶剂体积为2 mL。

### 2.3 复溶液比例优化

本文对比了分别使用20%、30%、40%、50%、60%、70%和80%甲醇水溶液作为复溶液时，各目标物的绝对回收率情况。结果表明，使用20%甲醇水溶液进行复溶时，BPF、BPA、BPB和BPS的绝对回收率接近100%，说明已基本复溶完全，而此时BPPH、BPG和BPM的绝对回收率经计算仅为18.1%、34.8%和47.7%。当甲醇比例升高至50%时，BPPH、BPG和BPM的绝对回收率分别增至63.3%、63.2%和86.4%，并且当甲醇比例进一步升高时趋于平稳。这是因为目标物的非极性较大，在水相比例较高时，其溶解度变小，导致绝对回收率降低；而在有机相比例较高时，其溶解度增大，绝对回收率提高。综合考虑高比例有机相复溶液容易对较早出峰的极性化合物产生溶剂效应，同时结合非极性化合物的复溶效果，最终选择50%甲醇水溶液作为氮吹后的复溶液。

### 2.4 基质效应与内标对应关系的确定

人体尿液成分复杂，当其中的干扰成分被一同提取出来时，可能会抑制或增强目标分析物的离子化程度，进而产生基质效应（matrix effect，ME）。当目标分析物在纯溶剂与基质中的响应存在较大差异时，会影响分析结果的准确性。本研究按1.3和1.4节方法对10份儿童混合尿样进行前处理和净化，将得到的复溶液作为样品基质，根据各目标化合物的本底含量（表2）和检出限（LOD，表3），从0.2、0.5、1、2、5和10 μg/L中选取3个质量浓度，配制对应的基质匹配标准曲线，并通过ME=（基质匹配标准曲线斜率/溶剂标准曲线斜率-1）×100%来评价基质效应。当|ME|<20%时，判定存在弱基质效应；当20%≤|ME|<50%时，判定存在中等基质效应；当|ME|≥50%时，判定存在强基质效应^［25］^。结果如表2所示，BPE、BPZ、BPG、BPM、BPAF和BPP、BPBP等7个目标化合物呈现弱基质效应，其余目标化合物均呈现中等基质效应。因此，为减少基质效应对样品测定结果准确性的影响，本方法采用内标法定量。

**表2:** 17种BPs的本底含量、基质效应和对应内标

Analyte	Background/（μg/L）	ME/%	Quantitative internal standard
BPS	0.009	43.3	BPS-D_8_
BPF	0.015	31.4	BPF-^13^C_6_
BPE	0.009	19.7	BPE-^13^C_6_
BPA	0.276	38.2	BPA-D_4_
BPB	<LOD	28.0	BPB-D_8_
BPAF	0.007	7.7	BPAF-^13^C_12_
BPC2	<LOD	32.0	BPAF-^13^C_12_
BPC	<LOD	22.9	BPB-D_8_
BPZ	<LOD	17.5	BPZ-^13^C_12_
BPAP	<LOD	22.0	BPAP-D_5_
BPM	<LOD	3.0	BPZ-^13^C_12_
BPG	<LOD	2.5	BPF-^13^C_6_
BPBP	<LOD	15.4	BPS-D_8_
BPP	<LOD	10.7	BPP-^13^C_4_
TCBPA	<LOD	33.4	BPZ-^13^C_12_
TBBPA	<LOD	27.1	TBBPA-D_4_
BPPH	<LOD	27.1	BPP-^13^C_4_

**表3:** 17种BPs的线性方程、线性范围、相关系数、检出限和定量限

Analyte	Linear equation	Linear range/（μg/L）	*r*	LOD/（μg/L）	LOQ/（μg/L）
BPS	*y*=0.09225*x*+0.02079	0.05-40	0.9995	0.023	0.056
BPF	*y*=0.19776*x*+0.01677	0.05-40	0.9999	0.054	0.145
BPE	*y*=0.09474*x*+0.00536	0.10-40	0.9999	0.035	0.096
BPA	*y*=0.11050*x*+0.00693	0.10-40	0.9999	0.489	0.986
BPB	*y*=0.48490*x*-0.01070	0.05-40	0.9998	0.002	0.005
BPAF	*y*=0.08970*x*+0.02855	0.20-40	0.9994	0.017	0.040
BPC2	*y*=0.00176*x*+0.00004	0.10-40	0.9999	0.029	0.097
BPC	*y*=0.10818*x*-0.00014	0.05-40	0.9999	0.007	0.022
BPZ	*y*=0.08527*x*+0.01414	0.05-40	0.9996	0.003	0.009
BPAP	*y*=0.09803*x*+0.02076	0.05-40	0.9997	0.003	0.010
BPM	*y*=0.00511*x*+0.00072	0.05-40	0.9994	0.006	0.019
BPG	*y*=0.09372*x*+0.03280	0.05-40	0.9986	0.025	0.084
BPBP	*y*=0.01528*x*+0.00178	0.05-40	0.9998	0.003	0.011
BPP	*y*=0.13654*x*-0.01992	0.05-40	0.9996	0.003	0.010
TCBPA	*y*=0.04376*x*+0.00368	0.05-40	0.9996	0.002	0.006
TBBPA	*y*=0.12805*x*-0.00288	0.05-40	0.9998	0.007	0.023
BPPH	*y*=0.04671*x*-0.01342	0.05-40	0.9987	0.017	0.057

*y*： peak area ratio of the target compound to the corresponding internal standard； *x*： mass concentration， μg/L.

在样品基质复杂、检出水平低且对准确度要求较高的内暴露检测工作中，同位素内标稀释法是确保样品准确定量的关键手段。然而，受限于同位素市场化的现状，往往难以保证每个目标化合物都能拥有与之对应的同位素内标。对于那些没有对应同位素内标的目标化合物，需要综合考虑样品前处理过程中的回收率以及基质效应这两个因素，从现有的内标中挑选出一个最为合适的作为其定量内标。本研究涉及的17种BPs中有10种BPs拥有一一对应的同位素内标，而另外7种BPs无对应的定量内标。在混合空白尿液中加入不同体积的混合标准溶液（加标水平设为0.1、0.2、0.5、1和5 μg/L）及30 μL内标混合溶液（50 μg/L），按1.3和1.4节方法进行前处理后上机分析。分别采用10种同位素内标来计算没有对应内标的7种BPs的相对回收率（经内标校正后的测定水平/实际加标水平），并从中选择在5个加标水平下平均相对回收率最接近100%的同位素标记物作为其最佳定量内标。由此得到的同位素内标对应关系见表2。

### 2.5 方法学验证

#### 2.5.1 线性范围、检出限和定量限

按1.2节方法配制系列质量浓度的混合标准工作溶液，按1.5节条件进样分析。以目标化合物与对应内标的峰面积之比（*Y*）对目标化合物的质量浓度（*X*，μg/L）绘制标准曲线。结合17种BPs的人群尿液暴露水平以及线性表现情况来确定合适的线性范围^［26，27］^。由表3可知，BPAF在0.2~40 μg/L范围内线性关系良好，BPA、BPE和BPC2在0.1~40 μg/L范围内线性关系良好，其余13种BPs在0.05~40 μg/L范围内线性关系良好，相关系数（*r*）均≥0.998 6。

通过对7个平行全流程空白样品的测定结果进行分析，发现BPF、BPE、BPA、BPS和BPAF均存在不同程度的背景污染。以这些目标化合物全流程空白样品测定值的均值加上3倍标准差作为其LOD，均值加上10倍标准差（SD）作为其定量限（LOQ）。对于在全流程空白样品测定过程中未检出的12种BPs，采用低水平加标法，即加入相当于3倍信噪比（*S/N*）对应质量浓度的目标化合物，随后以7个平行加标样测定值的3倍标准差作为该方法的LOD，10倍标准差作为LOQ^［28］^。17种BPs的LOD和LOQ如表3所示，LOD为0.002~0.489 μg/L，LOQ为0.005~0.986 μg/L。

#### 2.5.2 回收率和精密度

采用基质加标法考察本方法的准确度和精密度。选取低本底含量的儿童混合尿样作为加标基质，根据各目标化合物的本底含量值和LOD，设置低、中、高3个水平的加标回收试验，每个加标水平平行测定6次，并连续测定5 d，计算回收率和日内、日间精密度。其中，BPF、BPE、BPS、BPC2、BPG和BPPH的3个加标水平分别为0.2、0.5、1.0 μg/L，BPA的3个加标水平分别为0.5、1.0、5.0 μg/L，其余10种BPs的3个加标水平分别为0.1、0.2、0.5 μg/L。结果如表4所示，在3个加标水平下，17种BPs的回收率为61.1%~121.7%，日内和日间精密度分别为1.3%~11.2%和3.7%~19.0%。其中，TCBPA和BPPH由于缺少一一对应的同位素内标，二者的回收率分别为89.5%~121.7%和61.1%~69.2%，其余BPs的回收率为92%~118%，表明方法的准确度和精密度均良好。

**表4:** 3个加标水平下17种BPs的回收率、日内和日间精密度

Analyte	Spiked level/（μg/L）	Recovery/% （*n*=6）	Intra-day precision/% （*n*=6）	Inter-day precision/% （*n*=30）	Analyte	Spiked level/（μg/L）	Recovery/% （*n*=6）	Intra-day precision/% （*n*=6）	Inter-day precision/% （*n*=30）	
BPS	0.2	101.3	4.9	10.9	BPAP	0.1	101.9	1.9	7.7	
	0.5	96.6	2.2	6.5		0.2	95.7	7.4	8.5	
	1	98.5	4.1	7.3		0.5	100.2	4.6	8.3	
BPF	0.2	95.7	3	9.7	BPM	0.1	98.6	10.5	13.4	
	0.5	100.3	2.2	10.5		0.2	97.1	7.9	17	
	1	103	3.2	8		0.5	117.7	5.6	10.7	
BPE	0.2	91.7	3.7	7.1	BPG	0.2	98.4	6.1	16.1	
	0.5	95.7	2.1	8.2		0.5	98	6.5	13.5	
	1	97.5	4.5	7.5		1	99.6	7.2	13.7	
BPA	0.5	107.9	8.6	15.9	BPBP	0.1	96.8	6.8	10.9	
	1	103.8	3.4	8.6		0.2	98.9	8.7	14.2	
	5	104.3	4	7.9		0.5	95	4.5	12.2	
BPB	0.1	96.6	5.7	12.7	BPP	0.1	101.1	3.6	9	
	0.2	98.7	2.9	11.1		0.2	92.5	4.7	9.9	
	0.5	95.1	3.6	8.3		0.5	96.1	4	8.9	
BPAF	0.1	101.4	3.7	11.7	TCBPA	0.1	96.2	3.3	13.1	
	0.2	104.9	1.3	8.5		0.2	89.5	7.2	14.8	
	0.5	102.9	3.3	7.9		0.5	121.7	6.3	12.8	
BPC2	0.2	99.1	5	8.5	TBBPA	0.1	110.6	4.8	17.1	
	0.5	98.7	2.3	3.7		0.2	100.4	9.9	15.7	
	1	100.6	2	3.7		0.5	96	11.2	11.2	
BPC	0.1	97.4	7.3	19	BPPH	0.2	68.6	5.6	17.5	
	0.2	94.7	3.2	10.6		0.5	61.1	3.7	18.2	
	0.5	94.4	3	13		1	69.2	5.6	16.6	
BPZ	0.1	95.4	2.3	9.6						
	0.2	96.1	6.5	14.1						
	0.5	98	6.2	12.5						

#### 2.5.3 稳定性

通过监测加标样品在不同温度条件下待测物质含量的变化情况，能够为实际样品从采集到检测的时间安排提供指导。本研究选取低本底含量的儿童混合尿样，将其配制成17种BPs质量浓度分别为0.5 μg/L（低水平）和5 μg/L（高水平）的加标尿样，并分别置于4 ℃冷藏和-20 ℃冷冻条件下进行储存。对于冷藏的加标样品，分别在第0、1、3、5和7 d进行测定；对于冷冻的加标样品，则分别在第0、1、2、3和4周进行测定，每次测定结果均与各自第0 d测定的初始含量进行比较。研究结果显示，在4 ℃条件下储存7 d后，低水平加标尿液中的BPE、BPB、BPS、BPC、BPG和BPM的含量均比初始含量下降超过5%；高水平加标尿液中，BPE和BPA的含量下降超过5%。而在-20 ℃条件下储存4周后，低水平加标尿液中的BPE、BPA、BPB、BPP、TBBPA、BPC、BPC2、BPG、BPM和BPBP的含量均比初始含量下降超过5%；高水平加标尿液中，TBBPA、BPG、BPM和BPBP的含量下降超过5%。由此可见，尿液中含量较高的BPs稳定性相对更好，而长时间储存会对低水平BPs的检出产生影响。此外，在4 ℃冷藏至第3 d以及-20 ℃冷冻至第4周时，超过一半的BPs开始出现降解现象。因此，对于采样后暂存在4 ℃冷藏的尿样，应尽快进行测定；若无法尽快测定，则应置于-20 ℃保存，并在一个月内完成测定；若需进行更长期的保存，则应将尿样在-80 ℃条件下冻存。

#### 2.5.4 标准物质的检测

采用本方法对NIST的标准尿样SRM 3672（来自吸烟者）和SRM 3673（来自非吸烟者）进行测定，每组平行测定6次。经检测，这两种SRM中均仅含有BPA。对于SRM 3672，测定结果的均值为3.05 µg/kg，相对标准偏差（RSD）为3.5%，参考值为（3.05±0.16） µg/kg；对于SRM 3673，测定结果的均值为1.94 µg/kg，RSD为3.2%，参考值为（1.96±0.11） µg/kg。结果表明，测定值均处于参考值范围之内，且与参考值的中位数较为接近，这表明所建立的方法具有较高的准确度和良好的稳定性。

为检验所建立方法的可靠性，运用本方法参与了2023年德国外部质量评估计划（the German External Quality Assessment Scheme for Analyses in Biological Materials， G-EQUAS）的第71轮国际比对。测定项目包括BPA、BPS和BPF，涵盖了高、低两份不同含量的尿样（A和B），测定结果与参考值如表5所示，结果显示，两份尿液样本中的3种BPs的测定结果均处于参考值范围之内。这一结果进一步证实了本方法在测定人体尿液中痕量BPs时具备较高的灵敏性和准确性。

**表5:** G-EQUAS中3种BPs的测定结果与参考值对比

Analyte	Urine sample	Determination result/（µg/L）	Reference value/（µg/L）	Tolerance range/（µg/L）
BPA	A	1.16	1.21	0.82-1.60
	B	13.60	14.62	11.47-17.77
BPS	A	0.68	0.63	0.45-0.81
	B	3.32	3.30	2.67-3.93
BPF	A	1.66	1.68	1.32-2.04
	B	4.60	4.59	3.78-5.40

G-EQUAS： the German External Quality Assessment Scheme for Analyses in Biological Materials.

### 2.6 实际样品检测

采用本方法对50份随机尿样中的17种BPs进行了测定。在测定前，尿样被储存于-80 ℃的条件下。测定结果显示，11种BPs均有不同程度的检出，具体包括BPF、BPE、BPA、BPB、BPS、BPZ、BPAP、BPG、TCBPA、BPPH和TBBPA。其中，BPS和BPA的检出率最高，分别为98.0%和86.0%；其次是TCBPA，检出率为70.0%。此外，BPF、BPAP、BPE、BPB和TBBPA的检出率大于10%。在11种有检出的BPs中，BPA的检出含量最高，检出含量中位值为0.829 μg/L（LOD~8.117 μg/L）；其次是BPS，检出含量中位值为0.075 μg/L（LOD~10.781 μg/L）。本研究人体尿液样本中BPs的检出含量和检出率与相关生物监测研究报道相符^［29，30］^，这反映出人体对环境内分泌干扰物BPs暴露的普遍性。同时，该结果也提示BPS已成为我国生产和应用最为广泛的BPA替代品。

## 3 结论

本方法基于SLE-UHPLC-MS/MS技术，构建了一种可同时测定人体尿液中17种BPs的检测方法。该方法借助全自动SPE净化工作站，高效地对酶解后的尿液中的BPs进行富集与净化。通过优化前处理条件以及色谱、质谱条件，成功实现了17种BPs的分离、定性与定量分析。该方法简单快速、灵敏准确，在一定程度上规避了人工前处理可能引发的操作误差和交叉污染问题。该方法不仅提高了分析效率与通量，还展现出更好的重复性和准确性，同时降低了生物安全风险，能够满足人群队列研究中高通量样本测定的需求，为评估BPs在人群中的暴露水平以及制定相应的防治措施提供了有力的技术支持。随着未来BPs同位素标记物在市场上的普及，结合全自动前处理技术的分析方法有望在大规模人群生物监测与暴露评估研究中发挥重要作用，实现准确、灵敏且高通量的检测分析。
